# Sharp-Tailed Grouse Nest Survival and Nest Predator Habitat Use in North Dakota’s Bakken Oil Field

**DOI:** 10.1371/journal.pone.0170177

**Published:** 2017-01-12

**Authors:** Paul C. Burr, Aaron C. Robinson, Randy T. Larsen, Robert A. Newman, Susan N. Ellis-Felege

**Affiliations:** 1 Department of Biology, University of North Dakota, Grand Forks, ND, United States of America; 2 North Dakota Game and Fish Department, Dickinson, North Dakota, United States of America; 3 Department of Plant and Wildlife Sciences, Brigham Young University, Provo, UT, United States of America; 4 Monte L. Bean Life Sciences Museum, Brigham Young University, Provo, UT, United States of America; University of Alberta, CANADA

## Abstract

Recent advancements in extraction technologies have resulted in rapid increases of gas and oil development across the United States and specifically in western North Dakota. This expansion of energy development has unknown influences on local wildlife populations and the ecological interactions within and among species. Our objectives for this study were to evaluate nest success and nest predator dynamics of sharp-tailed grouse (*Tympanuchus phasianellus*) in two study sites that represented areas of high and low energy development intensities in North Dakota. During the summers of 2012 and 2013, we monitored 163 grouse nests using radio telemetry. Of these, 90 nests also were monitored using miniature cameras to accurately determine nest fates and identify nest predators. We simultaneously conducted predator surveys using camera scent stations and occupancy modeling to estimate nest predator occurrence at each site. American badgers (*Taxidea taxus*) and striped skunks (*Mephitis mephitis*) were the primary nest predators, accounting for 56.7% of all video recorded nest depredations. Nests in our high intensity gas and oil area were 1.95 times more likely to succeed compared to our minimal intensity area. Camera monitored nests were 2.03 times more likely to succeed than non-camera monitored nests. Occupancy of mammalian nest predators was 6.9 times more likely in our study area of minimal gas and oil intensity compared to the high intensity area. Although only a correlative study, our results suggest energy development may alter the predator community, thereby increasing nest success for sharp-tailed grouse in areas of intense development, while adjacent areas may have increased predator occurrence and reduced nest success. Our study illustrates the potential influences of energy development on the nest predator—prey dynamics of sharp-tailed grouse in western North Dakota and the complexity of evaluating such impacts on wildlife.

## Introduction

The United States has been gradually reducing its reliance on imported petroleum products by significantly increasing its own production [1]. This was primarily made possible through the advent of hydraulic fracturing in conjunction with horizontal drilling [2]. These technological developments, amongst others, have increased the amount of recoverable oil within formations and has made commercial-scale energy production possible throughout the nation [2,3]. North Dakota is currently one of the leading producers of oil in the U.S. [4], with more than 9,600 active oil wells at the end of 2013, approximately double that of eight years prior, with most development occurring in the Northwest [5].

The Bakken and Three Forks formations, which span the grasslands of western North Dakota into eastern Montana and southern Saskatchewan, contain most of the oil produced in the state [6,7]. The portion of the Bakken formation contained in North Dakota alone may sustain more than an estimated 38,000 oil wells and have the potential to impact more than one seventh of the state’s total land area [3]. This large-scale energy development results in economic growth and employment opportunities for the state, but also brings challenges in understanding and managing potential environmental impacts [8].

Various efforts have been made to understand how wildlife are affected while energy development continues to expand across the country [9]. Frequently this research has focused on species that pique public interest. For example, large mammals [10–14], sage-grouse [15], and various gamebirds and songbirds [16–19] all show varying signs of avoidance to structures related to energy developemt. In addition, lekking locations for certain grouse species appear to be sensitive to this activity [20]. However, little is currently known about the ecology of sharp-tailed grouse (*Tympanuchus phasianellus*) in the presence of energy development [8,16,20].

Sharp-tailed grouse (hereafter; sharp-tail) are a popular game bird throughout their range, and are recognized as an indicator species of grassland ecosystems health [8,21]. As such, this species is of particular concern for the U.S. Forest Service and North Dakota Game and Fish when making future prairie management decisions and understanding how landscape changes may influence grassland birds [21]. Sharp-tails are considered common throughout North Dakota, but immediate potential threats to their habitat include disturbances related to gas and oil development [8,16]. These disturbances have the potential to impact multiple aspects of sharp-tail ecology, both directly and indirectly. Common disturbances associated with gas and oil development include noise and light pollution, dust, traffic, road and housing development, and fragmentation of the landscape [3,9,16,18,22–24]. Possible influences from these disturbances on the nest survival of sharp-tails is an area of particular concern as nest survival is one important factor influencing reproductive success [25].

Similar to other prairie grouse species, sharp-tail nest failure is primarily caused by nest depredation [22,26–29]. Therefore, potential influences of energy development on the nest predator community are of equal concern when examining sharp-tail nest survival. Medium sized mammals, or meso-mammals, are common nest predators of ground nesting bird in western North Dakota [30], which primarily include coyotes (*Canis latrans*), striped skunks (*Mephitis mephitis*), American badgers (*Taxidea taxus*), raccoons (*Procyon lotor*), and red fox (*Vulpes vulpes*) [30,31]. In addition, these species often perform central roles in ecosystems as predators to a variety of other prey species [28,32–34]. However, research is lacking on how meso-mammals may be influenced by energy development. Any potential influences on the meso-mammal community may have indirect consequences for sharp-tails, as well as other prey species.

Our objectives for this study were to evaluate sharp-tail nest success and meso-mammal occupancy in northwestern North Dakota at two study areas varying in energy development. During the summers of 2012 and 2013 we used radio-marked hens and nest cameras to estimate daily nest survival of sharp-tails and camera scent stations to estimate meso-mammal occupancy rates at each site. We hypothesized that areas of increased energy development may have less favorable nesting habitat and experience increased disturbances which has been shown to elicit avoidance behaviors, lek abandonment, and reduced adult survival for grouse species (see [16]). We predicted nests within these areas may therefore have reduced nest success compared to its less developed counterpart. Similarly, we predicted the possibly more fragmented landscape of developed areas would facilitate predator travel and increase nest discovery efficiency thereby decreasing nest success as well [35,36].

## Study Areas

**Fig 1:**
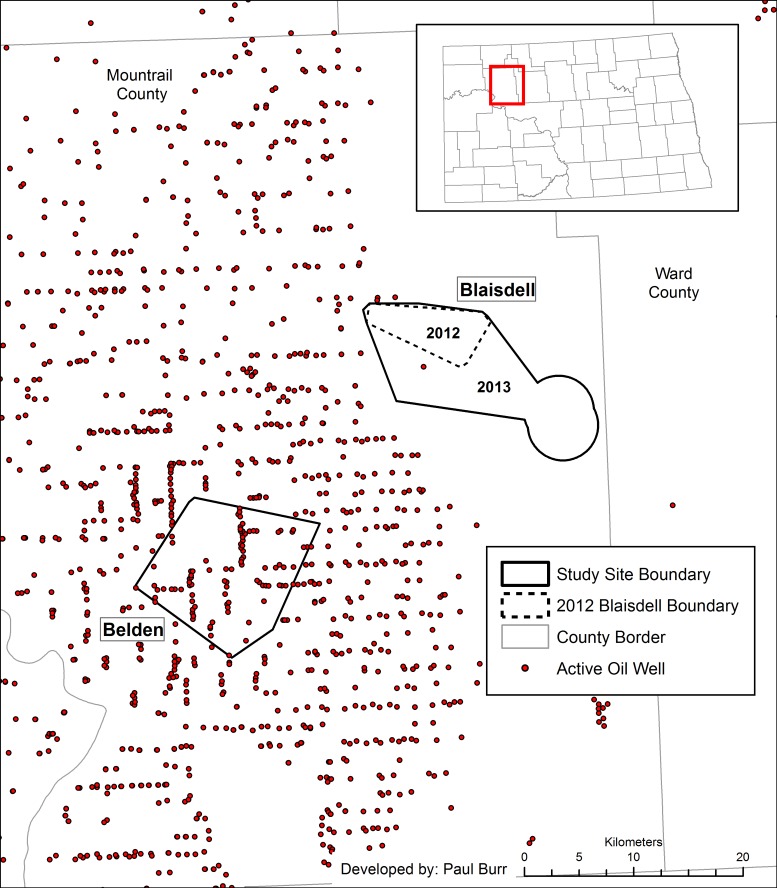
Two study areas established in Mountrail County of western North Dakota used to monitor sharp-tailed grouse nests and their nest predators in 2012 and 2013. Belden served as an area of intense oil development, whereas Blaisdell served as an area of minimal oil development for comparison. The dashed line within Blaisdell represents its boundary in 2012, and points represent active oil wells during our study years.

We selected two study areas, Belden and Blaisdell, with the goal of gathering data from areas with similar land use but differing levels of oil and gas intensity based on relative oil well densities (Fig 1). Study boundaries were constructed using 95% minimum convex polygons around sharp-tail nesting locations from 2010–2011 (A. C. Robinson, North Dakota Game and Fish Department, unpublished data). Study areas were located in Mountrail County, North Dakota and were approximately 15 kilometers apart from each other.

Belden covered 147.2 km^2^ (centroid: N 48.107922, W -102.393517), and was classified as intense oil activity with numerous active oil wells present within and around its boundary (Fig 1). Based on data from the North Dakota Industrial Commission [5], oil well density in Belden was 0.767 wells/km^2^ in August of 2012 and 0.950 wells/km^2^ in August of 2013. Belden was characterized as 61% grassland/hay land, 31% cropland, 6% wetland, and 2% trees/shrubs [37].

Blaisdell represented an area of minimal oil development and covered 38.7 km^2^ in 2012 (centroid: N 48.300744, W -102.130655), but was expanded to 158.3 km^2^ in 2013 (centroid: N 48.262096, W -102.077418) in order to create a more equal-sized study area and increase sample sizes. We expanded Blaisdell’s 95% minimum convex polygon by including sharp-tail nesting locations recorded during our first field season in 2012. We also included two additional lek locations discovered in 2012 to the polygon, and added a 3.22 km (2 mile) buffer around each of these leks to encompass potential nesting locations for sharp-tails using these leks ([38]; Fig 1). No active oil wells were within the 2012 boundary, but one oil well was within the extended 2013 boundary resulting in a density of 0.006 wells/km^2^ [5]. Although no active drilling of oil wells occurred within Blaisdell during this study, there was activity present around the study area, primarily to the west (Fig 1). Since it was still susceptible to some disturbances associated with oil development, we considered it an area of minimal development rather than no activity. The larger, 2013 Blaisdell polygon, was characterized as 44% grassland/hay land, 45% cropland, 11% wetland, and 0% trees/shrubs [37].

All study area land cover estimations, along with lands cover metrics used in subsequent analysis, were calculated using the U.S. Fish and Wildlife Service land cover layer [37]. We estimated classification accuracy of this data by randomly generating 200 points in each study area, proportional to the amount of each land cover (S1 Table). We compared these points to high resolution imagery (sub-meter) taken and mosaicked by the National Agriculture Imagery Program in 2012. Classification accuracy at Belden was 87.0%, and Blaisdell was 85.5%, giving a combined accuracy of 86.3%.

## Methods

### Field Methods

#### Nesting ecology of sharp-tailed grouse

We captured sharp-tail hens beginning in late April of 2012 and 2013 using walk-in funnel traps at leks [39]. Five leks were trapped at each study area, with the exception of Blaisdell in 2013 when we included two additional leks to expand its size (see study area). We checked traps four times a day, twice in the morning and twice in the evening. We removed sharp-tails from traps using a handheld net and placed them into a small fabric kennel before data collection. All grouse were individually aged (juvenile or adult), weighed, and banded. We fit hens with a VHF necklace style radio collar (10.7 or 16.0 grams; Advanced Telemetry Systems) and released all birds at the capture site immediately after data collection was over. We tracked hens weekly from May to July by radio telemetry using hand held, vehicle mounted, and fix winged aircraft mounted units and recorded all locations on GPS units. Once hens were found incubating a nest they were flushed to record the nest location and number of eggs. We confirmed nests remained active every 4–5 days using telemetry. If a hen was not found at her nest we then examined the nest bowl to determine if a depredation or hatching event occurred. A nest was considered successful if at least one egg hatched.

We monitored a subset of nests using 24-hour video surveillance nest cameras to accurately determine nest fates and identify nest predators. We opportunistically placed cameras at nests as they were found at the beginning of the nesting season to maximize the total number of nests video monitored. We were unable to monitor all nests due to our limited supply of cameras. Nests that received a camera later in the nesting season were systematically selected based on their general location within each study area. We placed cameras in this manner to ensure representative coverage throughout each study area, and all nests were subject to video monitoring, regardless of accessibility. Cameras were only placed on a nest once we believed incubation began (i.e. ≥ 11 eggs present; [38]). During camera installation, field technicians wore latex gloves to avoid leaving human scent. Cameras were clamped to a steel bar that was inserted into the ground approximately 0.5 m from the nest. A cord at ground level connected the camera to a digital video recorder (DVR) placed inside a waterproof box at least 30 m from the nest. The DVR and camera were powered by a 12 volt, 35 amp battery. Footage was recorded by the DVR unit and saved onto a 32-GB SD card. All camera equipment was camouflaged using paint and surrounding vegetation.

After installation of the camera was complete, we used telemetry to confirm the hen returned to its nest the following day. If the hen did not return we assumed camera presence influenced regular nesting and removed the camera. Batteries and memory cards were changed every 4–5 days to insure continuous recording. If the hen was absent from the nest at this time, we visually inspected the nest to determine if the nest was still actively being incubated. After a nest had been depredated or successfully hatched, all camera equipment was removed and placed at another active nest. All video footage was later reviewed to assess nest fates accurately, and all nest predators were identified to species if possible.

### Nest Predator Surveys

We conducted predator surveys using camera-scent stations during the sharp-tail nesting season (May–July; [38]) in 2012 and 2013. Each station consisted of a PC900 Hyperfire^TM^ Reconyx passive infrared field camera mounted approximately 1 m above the ground and placed approximately 5 m in front of a scent lure. During camera installation field technicians wore latex gloves to conceal human scent. Vegetation between the camera and scent lure was removed or reduced to create a clear line of sight for the camera. Each camera was set to take 3 consecutive photographs, 3 seconds apart. After the third photograph was taken the camera could not be triggered again for 5 minutes.

We deployed stations across each study area using a two-stage sampling design. First, we created a grid system with a cell size of 1 km by 1 km on each study area using ArcGIS (ESRI 2012, v10.1). A primary and secondary random point was generated for each grid cell along a habitat edge, as meso-mammals are thought to use such edges while traveling and foraging [35,36]. Edges were identified using the U.S. Fish and Wildlife Service [37] land cover layer in ArcGIS and were characterized as areas where water, grassland, agriculture, or trees/shrubs intersected. Each grid cell and its associated random point served as a potential location for one predator survey. Grid cells to be sampled were systematically chosen to ensure broad and even sampling across the two study areas. In some instances, selected grid cells or the primary point within the cell were not able to be sampled due to access limitations on private land. In such cases, we sampled using the secondary random point or the next closest grid cell not included in the sample.

We sampled over three separate periods spanning from 21 May to 30 July to increase coverage of the area. In 2012 each sample period lasted approximately 14 days. The three periods began on 21 May, 4 June, and 18 June, respectively. After discovering our detection probabilities were lower than expected in 2012, we increased the sample period length in 2013 [40]. Each sample period in 2013 lasted approximately 22 days and began on 24 May, 16 June, and 8 July, respectively.

Within the first half of each survey, we used a fatty acid scented predator disk (Pocatello Supply Depot, Pocatello, Idaho) to lure mammalian predators to the camera. To avoid predator acclimation to the scent of the predator disks, we replaced them half way through the survey with Caven’s “Violator 7” predator lure (Minnesota Trapline Products, Inc., Pennock, Minnesota) with the goal of maintaining predator interest and increasing detection probabilities. This second scent lure was placed inside of a hollowed golf ball that was mounted on a wooden dowel and staked into the ground. The golf ball served as a visual stimulus thought to elicit explorative behavior in meso-mammals such as coyotes when in unfamiliar environments [41,42]. In the event of major precipitation, we replaced scent lures to avoid scent being washed out. We identified animals in photos to species and recorded them per sampling occasion. We classified one sampling occasion as a 72 hour period (three calendar days). We omitted detections observed during the day of installation or termination of the camera-scent station as these did not encompass a full day.

All sampling procedures, including sharp-tail capturing, handling, and camera monitoring as well as predator scent station protocols were approved by the Institutional Animal Care and Use Committee of the University of North Dakota and strictly followed their procedures (#A3917-01, Protocol 1201–2). This work was done in collaboration with the North Dakota Game and Fish department, who served as a regulatory agency for our research. All efforts were made to minimize the amount of handling time and reduce the amount of stress on sharp-tails during the study. Permission to conduct research activities on private land was obtained directly from all land owners within our study areas. We also obtained permission from the North Dakota Game and Fish department for use of all state owned land, and from the U.S. Fish and Wildlife Service for federally owned land. This field study did not involve endangered or protect species throughout its duration.

### Data Analysis

#### Nesting ecology of Sharp-tailed grouse

We used video footage to identify nest predators, and modeled daily nest survival rates using Program MARK [43–46]. We included all nests monitored in both years, regardless of individual hen re-nests within or between years. There were 18 total re-nests (11% of all nests, 8 from Belden and 10 from Blaisdell) within a given nesting season used in the analysis. However, program MARK does not allow for random effects, so we first ran our analysis without including re-nest events, and then ran the same analysis while including re-nests for comparison. We chose to treat each of the 18 re-nests as individual nesting events if the results were similar, primarily to maintain a high sample size. We did not include any nests that appeared to fail due to abandonment caused by researcher disturbance or camera presence.

We explored the influence of multiple covariates in our daily nest survival models (S2 Table). Study area was included as a grouping variable, and year and nest camera presence were included as binary covariates. We hypothesized oil wells and roads may influence nest survival by potentially impacting local nest predator activities. However, due to model convergence issues, we could not include these variables as continuous data. This was likely the result of low variability associated with distance to nearest road, and the fact all Blaisdell nests were a considerable distance from the closest oil well. Therefore, we included Euclidean distance to the nearest active oil well and nearest road as categorical covariates. Nearest active oil well was classified as less than 450 m, 450–1,000 m, or greater than 1,000 m from the nest. Nearest road was classified as either less than 450 m or greater than 450 m from the nest. We selected these incremental distance categories relative to the approximate 450 m average movement distance of sharp-tail hens while laying and incubating eggs [47].

We included habitat composition around nest locations using multiple spatial scales in model construction. To classify composition, we used the U.S. Fish and Wildlife Service 30 m resolution land cover layer [37] and lumped land cover categories as either water, grassland, agriculture, or trees/shrubs in ArcGIS. We calculated percent composition for each category within a 50 m, 200 m, and 450 m buffer centered at the nest, again focusing on the 450 m buffer of habitat use by hens while incubating [47]. The 50 m buffer captured differences between nests at the microsite level, and the 200 m buffer was used as an intermediate measure. Edge density (m/km^2^) was also calculated at the 450 m extent with edges characterized as roads and where land cover types changed. We hypothesized this edge metric may influence survival as numerous mammalian nest predators exploit habitat edges when traveling and foraging for prey items [35,36,48,49]. We did not mix spatial extents when including habitat composition within models.

We based model selection on Akaike’s Information Criterion scores corrected for small sample sizes (AIC_c_) to determine which models had the most support [50]. We estimated daily nest survival rates (S), as well as individual covariate beta estimates (ß) using model averaging of all models constructed [50]. We back-transformed beta estimates to their respective odds ratio (OR) for interpretation. Odds ratio confidence intervals including 1.0 are not considered statistically significant, but may be biologically important if estimates are trending away from 1.0. For this study, we refer to these potentially important biological results as trending.

#### Nest predator surveys

We constructed single season occupancy models in program MARK to estimate predator occupancy [46]. Because our study included only two years of data, and not all survey sites were sampled in both years, we chose to include year as a covariate in our analysis rather than using a robust model design. Moreover, our goal was to determine if differences in species occurrence existed between study areas, rather than directly modeling changes in occupancy between years.

We developed a candidate set of biologically relevant models for all species detections pooled together. Specifically, a survey location was considered occupied if any target species was photographed at least once during a sampling occasion. We did not explore individual species occupancy models due to low detections. We used combinations of multiple covariates for occupancy and detection and compared these models using AIC_c_ (S3 Table; [50]). We first identified the best covariates that explained the detection parameter while keeping occupancy constant in our model construction. We explored the effects of year and sampling period (time of summer) as covariates (S3 Table). Year was a binary variable with 2012 serving as the baseline for comparison. We used dummy variables to model sampling period, where period one and period two were compared to the baseline of period three. We also evaluated if detection rates differed between individual occasions or between scent lures.

After determining which covariates best explained the detection parameter, we modeled covariates for the occupancy parameter. We examined the covariates of study area (Blaisdell as our baseline), year, Euclidean distance to nearest oil well (m), Euclidean distance to nearest road (m), and oil well density and habitat composition within a 500 m radius of the survey location (S3 Table). A 500 m buffer was chosen to limit the amount of overlap between neighboring survey locations while maintaining independence between sampling sites. We lumped habitat composition into similar land cover categories and classified them as water, grassland, agriculture, or trees/shrubs. We then calculated the percentage of area covered by each of these categories within the 500 m buffer around the survey locations in ArcGIS.

We model averaged estimates of occupancy (ψ), detection (*p*), and individual covariate beta’s (ß; [50]). Beta estimates were back-transformed to their respective odds ratios (OR) for interpretation as previously described for nest survival. We used model deviance to assess fit of models using the most general model with the distribution of deviance values obtained from 1,000 parametric bootstrapping replicates from program MARK [51]. Program MARK is unable to perform the bootstrap procedure with the incorporation of individual covariates [52] so we selected a general model without covariates for this step.

We tested for multicollinearity among all continuous variables by calculating Pearson’s correlation coefficients for both the nest survival and nest predator analyses. We took a conservative approach and did not use both covariates in the analysis if r^2^ > 0.3 [53]. We also tested for spatial autocorrelation to verify we did not violate the assumption of spatial independence by calculating Moran’s I in program Spatial Analysis in Macroecology (SAM Version 4.0, http://www.ecoevol.ufg.br/sam/, accessed 12 September 2013). To calculate this we used nest success (successful [0] vs. failed [1]) for the nest survival analysis and species detection (present [1] vs. not-present [0]) for the predator occupancy analysis. We assessed spatial autocorrelation by calculating Moran’s I values and associated p-values for each study area and year.

## Results

### Nesting Ecology of Sharp-Tailed Grouse

We monitored 163 sharp-tail nests across both study areas and years (S1 Dataset). Apparent nest success at Belden (i.e. intense gas and oil development area) was 62% based on 79 nest events across years, and 44% at Blaisdell (i.e. minimum gas and oil development area) based on 84 nest events across years (Table 1). Ninety of these nests were also monitored using nest cameras, with 42 deployed at Belden and 48 at Blaisdell, across years. Overall apparent nest success was 58.9% for nests monitored with cameras, 45.2% for those not monitored with cameras (Table 1). Eleven total nest abandonments occurred across both study areas and years after the hen was initially flushed or after nest camera installation. These were not included in any subsequent analysis.

**Table 1:** Summary of sharp-tailed grouse nests monitored in 2012 and 2013 in western North Dakota. Nests are delineated by study area, monitoring method, and categories of nest failures. Belden study area represents intense gas and oil development, whereas Blaisdell represents minimal development.

			Total Nests Monitored	Depredated	Hen Mortality	Cattle Trampling	Farm Machinery	Apparent Nest Success
All Nests			163	62	7	5	3	52.8%
By Study Area								
	Blaisdell		84	43	3	0	1	44.0%
	Belden		79	19	4	5	2	62.0%
By Monitoring Method								
	Nest Camera		90	29	4	3	1	58.9%
	Telemetry Only		73	33	3	2	2	45.2%
Combined								
	Blaisdell							
		Nest Camera	48	23	2	0	0	47.9%
		Telemetry Only	36	20	1	0	1	38.9%
	Belden							
		Nest Camera	42	6	2	3	1	71.4%
		Telemetry Only	37	13	2	2	1	51.4%

Seventy-seven nests (47.2%) failed across both study areas and years. Depredation was the leading cause of nest failures, accounting for 81% (n = 62) of all failed nests (Table 1). Belden had fewer depredations (n = 19, with 6 captured on camera) compared to Blaisdell (n = 44, with 24 captured on camera). Hen mortality accounted for 9% of all nest failures, followed by cattle trampling (6%), and farm machinery (4%; Table 1). Twenty-nine of the 37 nest failures captured on camera were depredation events. American badgers and skunks were the primary nest predators accounting for 30.0% (n = 9) and 26.7% (n = 8) of all recorded depredations, respectively. Raccoons were responsible for the third most depredations (16.7%, n = 5), all of which occurred at Blaisdell. Coyotes accounted for 10.0% of depredations (n = 3), followed by red fox (6.7%, n = 2). We observed two raptor depredations of eggs, one from a northern harrier (*Circus cyaneus*) and one species unidentifiable because of vegetation in front of camera.

We found evidence of correlation among percent grass and percent agriculture at each spatial extent (r^2^ = 0.95 for all extents). We hypothesized percent grass cover around the nest would have a larger influence on nest survival and therefore excluded percent agriculture from our analysis. In addition, we also excluded percent trees as there was extremely low variation among nest locations. In fact, 78.5% (128 out of 163) of the nest locations had 0% trees within the largest spatial extent of 450m, and average percent trees for all nests was lower than 1.4% at each spatial extent. Moran’s I correlograms revealed no evidence of spatial autocorrelation (p > 0.05) among nest fates (i.e., success or failure).

We constructed 59 models to evaluate sharp-tail nest survival. Models sets with, and without re-nests included produced extremely similar results. We are therefore only reporting models with re-nests included here. Study area and camera presence were in the top ranked model as the best predictors describing daily nest survival rates (Table 2). These two covariates were also included together in the next top 11 models, containing 89% of all AICc weight. Additionally, study area and camera presence were included in combination or alone with a combination of other covariates in models containing over 99% of all AICc weight. Belden nests were 1.95 times more likely to succeed than nests at Blaisdell (Table 3). Model-averaged daily nest survival was 0.975 (95% CI = 0.963–0.984) in Belden, and 0.955 (95% CI = 0.937–0.967) in Blaisdell. Nest survival over the average 23 day incubation period of sharp-tails was 55.9% at Belden and 34.7% at Blaisdell. Camera monitored nests were 2.03 times more likely to succeed than non-camera monitored nests (Table 3).

**Table 2:** Models within two ΔAIC_c_ scores from the highest ranked daily nest survival (S) model constructed for sharp-tailed grouse in western North Dakota, 2012–2013. Relative model weight (*w*), likelihood estimate (L), parameter count (K), and deviance are also displayed. Null deviance for nest survival = 531.17.

Model	AIC_c_	ΔAIC_c_	*w*	L	K	Deviance
S(Study Area + Nest Cam)	520.29	0.00	0.16	1.00	3	514.28
S(Study Area + Nest Cam + 50m Grass)	520.77	0.48	0.13	0.79	4	512.75
S(Study Area + Nest Cam + 200m Grass)	521.67	1.38	0.08	0.50	4	513.65
S(Study Area + Nest Cam + 50m Water)	521.80	1.51	0.08	0.47	4	513.78
S(Study Area + Nest Cam + 450m Grass)	521.89	1.60	0.07	0.45	4	513.88
S(Study Area + Nest Cam + Year)	521.99	1.70	0.07	0.43	4	513.98
S(Study Area + Nest Cam + DistRoad)	522.06	1.77	0.07	0.41	4	514.04
S(Study Area + Nest Cam + 200m Water)	522.17	1.88	0.06	0.39	4	514.15
S(Study Area + Nest Cam + 450m Water)	522.24	1.95	0.06	0.38	4	514.23

Covariate description: Study Area, study area as a grouping variable; Nest Cam, nest camera presence on the nest; DistRoad, distance to nearest road from the nest; Year, year as a categorical variable; Also shown are specific habitat compositions within the displayed distance from the nest.

**Table 3:** Model-averaged beta (β), standard error (SE), and 95% confidence interval (CI) estimates from Program MARK for all covariates included in the sharp-tailed grouse daily nest survival analysis from North Dakota, 2012–2013. Associated odds ratios (OR) and 95% confidence intervals are also calculated for interpretation of results.

	β	β	β	β	Odds Ratio	OR	OR
Model covariate	Estimate	SE	95% LCI	95% UCI	(OR)	95% LCI	95% UCI
Intercept	2.566	0.350	1.879	3.253			
Study Area*	0.669	0.267	0.147	1.191	1.952	1.158	3.292
Camera*	0.708	0.237	0.244	1.172	2.029	1.276	3.227
Year	-0.128	0.241	-0.601	0.346	0.880	0.548	1.413
Distance to Road (m)	-0.109	0.249	-0.596	0.379	0.897	0.551	1.460
Distance to Well (0-450m)	-0.091	0.616	-1.298	1.117	0.913	0.273	3.056
Distance to Well (451-1000m)	0.043	0.556	-1.047	1.134	1.044	0.351	3.107
50m Grass %	0.005	0.004	-0.003	0.013	1.005	0.997	1.013
50m Water %	-0.023	0.031	-0.084	0.037	0.977	0.919	1.038
200m Grass %	0.004	0.005	-0.005	0.013	1.004	0.995	1.013
200m Water %	-0.014	0.040	-0.092	0.064	0.986	0.912	1.067
450m Grass %	0.004	0.006	-0.007	0.015	1.004	0.993	1.015
450m Water %	0.009	0.037	-0.064	0.082	1.009	0.938	1.085

* Indicates statistically significant terms (i.e., odds ratio did not overlap 1.0)

Other covariates contained in candidate models within 2 ΔAIC_c_ scores from the top model included habitat composition metrics from each spatial extent, year, and distance to roads (Table 2). However, model-averaged beta estimates and associated odds ratios revealed these covariates had little influence on daily nest survival (Table 3). All models containing the covariate of edge density within 450 m failed to converge and were not reported.

### Nest Predator Surveys

We deployed 62 scent-stations in 2012, and increased this to 101 in 2013 to expand study area coverage, yielding 940 separate trap occasions over both areas and years (S1 Dataset). Fifty of the original 62 survey locations were resampled in 2013. Nest predator detections are summarized in Table 4.

**Table 4:** Mammalian nest predator detections recorded from camera scent-stations deployed in 2012 and 2013 at two study areas in western North Dakota. Numbers listed for each species represent the number of stations a species was detected at least one time.

	2012		2013		
	Belden	Blaisdell	Belden	Blaisdell	
	(Intense Development)	(Minimal Development)	(Intense Development)	(Minimal Development)	Total
Number of Stations	33	29	51	50	163
Sampling Occasions (72 hour period)	132	116	349	343	940
Coyote	5	8	22	26	61
American badger	0	6	7	13	26
Raccoon	2	3	5	16	26
Striped skunk	1	4	5	9	19
Red fox	0	1	0	2	3

We found no evidence of lack of fit for any of the general models that successfully converged. Percent agriculture and percent grassland were the only covariates exhibiting multicollinearity (r^2^ = 0.912). We used grassland habitat to examine composition on predator occurrence. We also excluded percent trees as there was extremely low variation among the scent stations similar to our nest survival analysis. We found no evidence of spatial autocorrelation for any of the individual species detections or when species were lumped together (p > 0.05). Due to model convergence issues, we had to omit the covariates of distance to nearest active oil well and nearest road.

Sampling period and year were the best explanatory variables for detection while holding occupancy constant. We constructed 24 models exploring various relationships between covariates and occupancy. Model averaged estimates for daily detection probabilities for the predator community were 0.286 (95% CI = 0.231–0.347). Detections were estimated to be 1.2 times greater during the first sampling period compared with sampling period three, but the difference was not statistically significant. We found sampling period two was 1.9 times less likely to detect a meso-mammal compared to period three, and year did not show a strong influence on detection (Table 5).

**Table 5:** Model-averaged beta (β), standard error (SE), and 95% confidence interval (CI) estimates for all covariates included within the occupancy and detection parameter based on all models constructed. Associated odds ratios (OR) and 95% confidence intervals were also calculated for result interpretation. Models include detections from coyotes, American badger, raccoons, skunks, and red fox.

		β	β	β	β	Odds Ratio	OR	OR
Model parameter		Estimate	SE	95% LCI	95% UCI	(OR)	95% LCI	95% UCI
Occupancy								
	Intercept	0.770	0.958	-1.108	2.649			
	Study Area*	-1.942	0.819	-3.547	-0.338	0.143	0.029	0.713
	Year*	1.872	0.593	0.709	3.035	6.503	2.032	20.808
	Percent Grass	-0.007	0.011	-0.029	0.015	0.993	0.972	1.015
	Percent Water	0.161	0.096	-0.027	0.348	1.174	0.973	1.417
	Well Density	0.039	0.206	-0.365	0.442	1.040	0.694	1.557
Detection								
	Intercept	-0.748	0.390	-1.513	0.017			
	Period 1	0.198	0.233	-0.257	0.654	1.219	0.773	1.924
	Period 2*	-0.633	0.261	-1.144	-0.122	0.531	0.319	0.886
	Year	-0.057	0.338	-0.718	0.605	0.945	0.488	1.831

* Indicates statistically significant terms (i.e., odds ratio did not overlap 1.0)

Study area and year appeared in the top eight models, which contained 95.5% of the total AICc weight (Table 6). Based on model-averaged estimates, we found Belden was 6.9 times less likely to be occupied by meso-mammals than Blaisdell and occupancy was 6.5 times greater in 2013 compared to 2012 (Table 5). Occupancy for the predator community as a whole was 0.896 (95% CI = 0.647–0.976) for Blaisdell, and 0.574 (95% CI = 0.356–0.767) for Belden. No other single covariates used in the analysis showed a significant trend on occupancy (Table 5).

**Table 6:** Candidate models within two AIC_c_ scores from the highest ranked model constructed for occupancy analysis in Program MARK. Relative model weight (*w*), likelihood estimate (L), parameter count (K), and deviance are also displayed. Species used in analysis include coyotes, American badger, raccoons, skunks, and red fox detected in western North Dakota, 2012–2013. Null deviance for nest predator occupancy = 928.46.

Model	AIC_C_	ΔAIC_c_	*w*	L	K	Deviance
p(P1 + P2 + Year) Ψ(Study Area + Year)	906.86	0.00	0.30	1.00	7	892.13
p(P1 + P2 + Year) Ψ(Study Area + PerWater + Year)	907.56	0.71	0.21	0.70	8	890.63
p(P1 + P2 + Year) Ψ(Study Area + PerGrass + Year)	908.32	1.46	0.14	0.48	8	891.38

Occupancy (Ψ) and detection (p) covariate description: Study Area, study area as a grouping variable; P1, survey period from 20 May to 18 June; P2, survey period from 19 June to 8 July; Year, year as a categorical variable; PerWater, percent water habitat within 500 meters; PerGrass, percent grass habitat within 500 meters.

## Discussion

This study is one of the first to simultaneously examine differences in both predator and prey relative to high and low levels of energy development. Although only correlative, we found a relationship between predator occurrence and nest survival relative to our study sites. Based on current literature we originally expected the direction of this relationship to be opposite of our findings. Instead, we observed higher meso-mammal occupancy and lower daily nest survival rates at our area of minimal gas and oil development (i.e., Blaisdell), and the opposite was observed at the adjacent area of intense gas and oil development (i.e., Belden). We hypothesize energy development in North Dakota is indirectly influencing sharp-tail nest success by altering local predator activity.

A number of studies have examined the effects of energy development on multiple ecological aspects of prairie grouse [54]. Most of these have reported overall negative effects such as potential reduced yearling and adult survival rates [20,55], behavioral avoidance of infrastructure [20,22,56–58], and reduced lek attendance [20,59–62]. However, sharp-tailed grouse nest success was found to be similar in areas with and without gas and oil development in the Little Missouri National Grasslands of North Dakota [63]. Similarly, no difference in nest success of greater sage-grouse was found between disturbed and undisturbed areas in Wyoming [56], and evidence of higher nest success for Greater prairie-chickens (*Tympanuchus cupido*) was found within more developed areas of Kansas [64]. In our study, apparent nest success at Belden was comparable to that reported elsewhere for sharp-tails, whereas Blaisdell was slightly lower [63,65–67].

Previous work has found a negative impact of human built infrastructure on mammalian abundance [68]. Gas and oil development introduces a variety of infrastructure such as access roads, buildings, camp sites, drill pads, and power lines [69]. Meso-mammals may be avoiding such structures and seeking out other habitat. In addition, areas under development experience large increases of vehicle traffic associated with the construction, drilling, and maintenance of oil wells [24]. This increase may reduce meso-mammal occurrence either by eliciting an avoidance behavior, or directly through mortality caused by vehicle collisions. One primary cause of mortality for many meso-mammal species is vehicle collisions [70–74]. Likewise, energy development produces high levels of chronic noise which can negatively impact a variety of different taxa [23,75]. Meso-mammals may be responding to some or all of these factors to different degrees, resulting in the reduced occurrence observed at our intense energy development study area.

Neither distances to active oil well or road were good predictors of sharp-tail daily nest survival, and were inestimable for meso-mammal occupancy. Generally, there was little variation in distance to roads among nests or predator scent-stations. This was most likely a result of the grid system for roads that exists across our study areas with roads located approximately 1.6 km (1 mile) apart. Further, much of the well pad development occurred directly along these roads. Although not captured in our analysis, we did notice substantially more vehicle traffic across our entire Belden study area than the Blaisdell site. This may have underlying impacts on the predator community, nest site selection, and hen stress, all of which may affect nest success and other demographic rates. Distance to active oil wells was most likely correlated with study area, as all nests except one in Blaisdell were greater than 1,000 m from an oil well.

The process of gas and oil development can commonly be broken down into four general stages: exploration, drilling, production, and abandonment. During the two years this study took place, we worked in an area dominated by the drilling stage, which includes the active construction and drilling of oil wells. These activities are often blamed for the fragmentation of landscapes through the introduction of roads, well pads, buildings, power lines, and other infrastructure [3,69,76]. Eventually this area is expected to be saturated with wells and will then be in the production stage, at which point human presence is only required for regular maintenance and inspection. This progression will result in a landscape left altered to a certain degree, but likely experiencing less disturbance than occurred during our study. Such fragmentation and the associated habitat edges are often exploited by predators when foraging and can be linked to decreased nest survival of ground nesting birds [35,36,77]. If disturbances associated with gas and oil are currently displacing predators and reducing nest depredations, it is possible as energy development progresses that predators may ultimately return and reduce nest success below natural variation in the newly fragmented landscape. This idea illustrates the importance of future research continuing to assess the impacts on wildlife as the dynamic process of gas and oil development progresses through each stage.

Unlike other studies (i.e., [38,47,49,77]), habitat composition had little influence in our daily nest survival analysis. For example, the habitat composition of agriculture has been found to be particularly influential on sharp-tail nest survival at rather broad extents (i.e. 1,600 m) [47]. We did not measure such a large extent as the nests we monitored were spatially clumped together and larger buffers would have resulted in substantial overlap. Overall, composition within 450 m of nests differed little between study areas (S4 Table). Similarly, no spatial covariates measured at the 500m scale extent used in our meso-mammal analysis were strong predictors of occupancy. Our findings of the importance of study area as a covariate may be indicative of the importance of larger spatial scales for both sharp-tails and meso-mammals, or that study area as a factor better represented the composite effects of habitat composition and disturbances than any linear combination of individual factor included as independent covariates.

Similar to other prairie grouse species, we found depredation to be the leading cause of nest failure for sharp-tails [22,26–29], and like other ground-nesting species, most were depredated by meso-mammals [30]. We confirmed the identity of primary nest predators for sharp-tails in western North Dakota using nest cameras, avoiding any potential bias of predator identification from sign left at the nest [26,78,79]. American badgers and skunks were responsible for the most depredations captured on camera at either site. Unfortunately, due to the low sample size of depredated nests recorded at our Belden site, we could not confidently make inferences regarding differences in nest predator frequencies between study areas. We observed two instances of raptors depredating eggs (not just hens). We did not observe any other avian nest predators such as members of the Corvidae family, which have been reported widely for ground nesting, grassland bird species [26,30,47,80]. Unfortunately, we do not have corvid abundance data in our study area to explain this lack of depredations. However, anecdotally we did observe some crows (*Corvus brachyrhynchos*) and magpies (*Pica hudsonia*), but only in very small numbers. Given the prairie landscape and few trees, we would not expect a high abundance of these species. This potential low abundance may explain the lack of Corvidae depredations, something that is commonly reported for grassland nesting bird species.

We observed a positive influence on daily nest survival with the presence of nest cameras. Similarly, a meta-analysis also found an overall positive effect of nest cameras on daily nest survival for a number of monitored avian species [81]. We believe two possible explanations, or a combination of each, could be driving this result. First, predators may be avoiding the novel structures of nest camera systems. If this is the case, then nest cameras may directly increase nest success by eliciting predator avoidance. Secondly, we generally deployed nest cameras later in incubation due to logistical restrictions, which may therefore be biasing our result as nests farther along in incubation are more likely to succeed [44]. Similar findings have been reported for the monitoring of greater sage-grouse nests [26,82].

Meso-mammal occupancy was consistently greater in the year 2013 compared to 2012. These findings agree with North Dakota’s annual rural mail carrier survey of furbearer species [83]. These surveys encompass large geographical regions of the state and are primarily used to evaluate trends in species populations. Coyotes, striped skunks, red fox, and raccoons all showed increases in the number of observations per 1,000 miles between 2012 and 2013 in our study region, whereas badgers showed only a slight six percent drop [83]. These reports also generally do not indicate differences in predator communities existed between the two adjacent study areas historically.

Time of summer consistently influenced our ability to detect meso-mammals, and is most likely linked to species activity patterns relating to their reproductive ecology (see [31]). Detection probabilities were greatest during the beginning of summer. With the exception of badgers, the meso-mammals observed here generally begin to breed at this time, and activity levels typically increase for mate selection and foraging purposes. Detection probabilities were lowest during the mid-summer when male activity generally decreases once the breeding season concludes. Female’s activity also decreases as the gestation period begins and families are typically restricted to dens as nursing takes place.

Ecological impacts of energy development have received considerable attention in recent years over the concern for the management and conservation of wildlife and their habitats. Here, we found a negative correlation with the meso-mammal community and a positive correlation with sharp-tail nest success and energy development at areas with similar habitat composition. However, the fast pace and large scale of gas and oil development occurring in western North Dakota makes before and after studies of the impacts on wildlife extremely difficult to conduct and causation problematic to establish. Further, given our lack of spatial replication at the study area level we were unable to evaluate the generality of the associations we observed. Similarly, additional factors such as chick and hen survival also have direct impacts on sharp-tail population dynamics and require further investigation.

Wildlife managers need to recognize that the process of gas and oil development is very dynamic, and each phase has differing implications for wildlife populations. Early stages of development include increased disturbances created from machinery, traffic, and human presence. Later stages of development have reduced human presence but may result in a landscape left altered. Such habitat changes may result in altered predator community dynamics that have potential impacts on local prey species found in areas of energy development. We estimated higher sharp-tail nest success at our high intensity study area and greater predation rates at the low intensity study area, demonstrating direct and indirect influences of energy development on wildlife populations. Development will continue to create significant habitat change which may influence wildlife as energy demands continue to increase. Continued research on this subject will ultimately help to understand these processes as well as mitigate impacts on local ecosystems.

## Supporting Information

S1 Table Classification accuracy estimated for the U.S. Fish and Wildlife Service land cover layer used in this study for two study areas established in Mountrail County, North Dakota. Accuracy was calculated by randomly generating 200 reference points in each study area, proportional to the amount of each land cover type, and comparing them to high resolution imagery.
(DOCX)

S2 Table Explanatory covariates used for evaluating sharp-tailed grouse daily nest survival rates in western North Dakota, 2012–2013. (DOCX)

S3 Table Explanatory covariates used for analyzing occupancy and detection rates of the mammalian nest predator community in western North Dakota, 2012–2013. (DOCX)

S4 Table Mean habitat composition within 450 meters of sharp-tailed grouse nests monitored at Belden and Blaisdell. (DOCX)

S1 Dataset Sharp-tailed grouse nest data, nest predator occupancy data, and all accompanied covariate data used for analysis. (XLSX)
