# Supplementary material for: Sharp-Tailed Grouse Nest Survival and Nest Predator Habitat Use in North Dakota’s Bakken Oil Field
Source: PLoS One. 2017 Jan 12;12(1):e0170177. doi: 10.1371/journal.pone.0170177 (PMC5231376; doi:10.1371/journal.pone.0170177)
Supplement: S1 Table — Accuracy was calculated by randomly generating 200 reference points in each study area, proportional to the amount of each land cover type, and comparing them to high resolution imagery. (DOCX) [file pone.0170177.s001.docx]

**S1 Table.** **Classification accuracy estimated for the U.S. Fish and Wildlife Service land cover layer used in this study for two study areas established in Mountrail County, North Dakota.** Accuracy was calculated by randomly generating 200 reference points in each study area, proportional to the amount of each land cover type, and comparing them to high resolution imagery.

| Belden | | | | | |
| --- | --- | --- | --- | --- | --- |
|  | Reference Data | | | |  |
|  | Water | Grass | Crop | Trees | Total |
| Water | 9 | 0 | 1 | 0 | 10 |
| Grass | 3 | 108 | 6 | 1 | 118 |
| Crop | 0 | 11 | 54 | 0 | 65 |
| Trees | 0 | 2 | 0 | 3 | 5 |
| Other | 0 | 1 | 1 | 0 | 2 |
| Total | 12 | 122 | 62 | 4 | 200 |
| Accuracy | 0.75 | 0.89 | 0.87 | 0.75 | 0.870 |
| Blaisdell | | | | | |
|  | Reference Data | | | |  |
|  | Water | Grass | Crop | Trees | Total |
| Water | 15 | 1 | 1 | 0 | 17 |
| Grass | 5 | 84 | 15 | 0 | 104 |
| Crop | 2 | 3 | 72 | 0 | 77 |
| Trees | 0 | 0 | 0 | 0 | 0 |
| Other | 0 | 2 | 0 | 0 | 2 |
| Total | 22 | 90 | 88 | 0 | 200 |
| Accuracy | 0.68 | 0.93 | 0.82 | na | 0.855 |
