# Supplementary material for: Sharp-Tailed Grouse Nest Survival and Nest Predator Habitat Use in North Dakota’s Bakken Oil Field
Source: PLoS One. 2017 Jan 12;12(1):e0170177. doi: 10.1371/journal.pone.0170177 (PMC5231376; doi:10.1371/journal.pone.0170177)
Supplement: S2 Table — (DOCX) [file pone.0170177.s002.docx]

**S2 Table.** **Explanatory covariates used for evaluating sharp-tailed grouse daily nest survival rates in western North Dakota, 2012–2013.**

| Covariate | Data Type | Description |
| --- | --- | --- |
| Area | Categorical | Study area: Belden or Blaisdell |
| Cam | Categorical | Presence or absence of a nest camera on nest |
| Year | Categorical | Study year: 2012 or 2013 |
| DistRoad | Categorical | Distance to nearest road: 0–450m, > 450m |
| DistWell | Categorical | Distance to nearest oil well: 0–450m (DistWell-1), 451m–1,000m (DistWell-2), or > 1,000m |
| 50Grs | Continuous | Percent grass within 50 meters of the nest |
| 50 Ag***** | Continuous | Percent agriculture within 50 meters of the nest |
| 50Wtr | Continuous | Percent water within 50 meters of the nest |
| 50Tr***** | Continuous | Percent Trees/shrubs within 50 meters of the nest |
| 200Grs | Continuous | Percent grass within 200 meters of the nest |
| 200 Ag***** | Continuous | Percent agriculture within 200 meters of the nest |
| 200Wtr | Continuous | Percent water within 200 meters of the nest |
| 200Tr***** | Continuous | Percent Trees/shrubs within 200 meters of the nest |
| 450Grs | Continuous | Percent grass within 450 meters of the nest |
| 450Ag***** | Continuous | Percent agriculture within 450 meters of the nest |
| 450Wtr | Continuous | Percent water within 450 meters of the nest |
| 450Tr***** | Continuous | Percent Trees/shrubs within 450 meters of the nest |
| 450Edge***** | Continuous | Edge density within 450 meters of the nest (m/km^2^) |

* Covariates not used in the analysis due to correlation or problems with model convergence.
