# Supplementary material for: Sharp-Tailed Grouse Nest Survival and Nest Predator Habitat Use in North Dakota’s Bakken Oil Field
Source: PLoS One. 2017 Jan 12;12(1):e0170177. doi: 10.1371/journal.pone.0170177 (PMC5231376; doi:10.1371/journal.pone.0170177)
Supplement: S3 Table — (DOCX) [file pone.0170177.s003.docx]

**S3 Table.** **Explanatory covariates used for analyzing occupancy and detection rates of the mammalian nest predator community in western North Dakota, 2012–2013.**

| Covariate | Data Type | Description |
| --- | --- | --- |
| Detection Parameter |  |  |
| Year | Categorical | Study year: 2012 or 2013 |
| Sample period (P) | Categorical | Corresponding to the time of summer camera-scent stations were deployed: 20 May – 18 June (P1), 19 June – 8 July (P2), or 9 July – 29 July. |
| Time (t) | Categorical | Detection was allowed to vary between sampling occasions |
|  |  |  |
| Occupancy Parameter |  |  |
| Area | Categorical | Study area: Belden or Blaisdell |
| Year | Categorical | Study year: 2012 or 2013 |
| DistWell***** | Continuous | Distance to nearest active oil well (m) |
| DistRoad***** | Continuous | Distance to nearest road (m) |
| WellDens | Continuous | Active oil well density within 500m of the camera-scent station (wells/km^2^) |
| PerGrass | Continuous | Percent grass within 500 meters of the camera scent station |
| PerWtr | Continuous | Percent water within 500 meters of the camera scent station |
| PerAg | Continuous | Percent agriculture within 500 meters of the camera scent station |
| PerTree | Continuous | Percent trees/shrubs within 500 meters of the camera scent station |

* Covariates not used in the analysis due to correlation or problems with model convergence.
