# Supplementary material for: Sharp-Tailed Grouse Nest Survival and Nest Predator Habitat Use in North Dakota’s Bakken Oil Field
Source: PLoS One. 2017 Jan 12;12(1):e0170177. doi: 10.1371/journal.pone.0170177 (PMC5231376; doi:10.1371/journal.pone.0170177)
Supplement: S4 Table — (DOCX) [file pone.0170177.s004.docx]

**S4 Table.** **Mean habitat composition within 450 meters of sharp-tailed grouse nests monitored at Belden and Blaisdell.**

|  | Belden (n=79) | | Blaisdell (n=84) | |
| --- | --- | --- | --- | --- |
| 450 meter buffer | Mean | Standard Deviation | Mean | Standard Deviation |
| Grass | 66.68 | 22.95 | 71.11 | 19.02 |
| Agriculture | 29.00 | 23.45 | 20.79 | 18.73 |
| Water | 1.59 | 1.65 | 8.07 | 4.42 |
| Trees | 2.71 | 4.72 | 0.02 | 0.13 |
